# Mechanical Behavior of Steel Fiber-Reinforced Concrete Beams Bonded with External Carbon Fiber Sheets

**DOI:** 10.3390/ma10060666

**Published:** 2017-06-17

**Authors:** Viktor Gribniak, Vytautas Tamulenas, Pui-Lam Ng, Aleksandr K. Arnautov, Eugenijus Gudonis, Ieva Misiunaite

**Affiliations:** 1 Research Laboratory of Innovative Building Structures, Vilnius Gediminas Technical University (VGTU), Sauletekio Av. 11, LT-10223 Vilnius, Lithuania; Vytautas.Tamulenas@vgtu.lt (V.T.); irdngpl@gmail.com (P.-L.N.); Eugenijus.Gudonis@vgtu.lt (E.G.); Ieva.Misiunaite@vgtu.lt (I.M.); 2 Department of Civil Engineering, The University of Hong Kong, Pokfulam, Hong Kong, China; 3 Institute of Polymer Mechanics, University of Latvia, Aizkraukles Str. 23, LV-1006 Riga, Latvia; Alexander.Arnautov@pmi.lv

**Keywords:** adhesive-mechanical connection, debonding failure, external CFRP sheets, mechanical testing, steel fibers reinforced concrete

## Abstract

This study investigates the mechanical behavior of steel fiber-reinforced concrete (SFRC) beams internally reinforced with steel bars and externally bonded with carbon fiber-reinforced polymer (CFRP) sheets fixed by adhesive and hybrid jointing techniques. In particular, attention is paid to the load resistance and failure modes of composite beams. The steel fibers were used to avoiding the rip-off failure of the concrete cover. The CFRP sheets were fixed to the concrete surface by epoxy adhesive as well as combined with various configurations of small-diameter steel pins for mechanical fastening to form a hybrid connection. Such hybrid jointing techniques were found to be particularly advantageous in avoiding brittle debonding failure, by promoting progressive failure within the hybrid joints. The use of CFRP sheets was also effective in suppressing the localization of the discrete cracks. The development of the crack pattern was monitored using the digital image correlation method. As revealed from the image analyses, with an appropriate layout of the steel pins, brittle failure of the concrete-carbon fiber interface could be effectively prevented. Inverse analysis of the moment-curvature diagrams was conducted, and it was found that a simplified tension-stiffening model with a constant residual stress level at 90% of the strength of the SFRC is adequate for numerically simulating the deformation behavior of beams up to the debonding of the CFRP sheets.

## 1. Introduction

**Figure 1:**
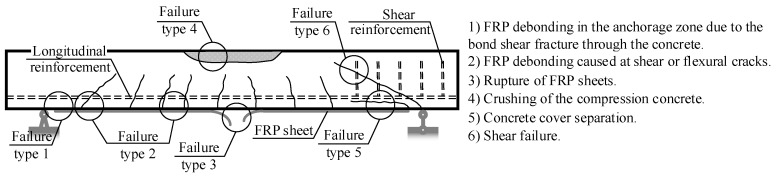
Types of failure of externally bonded reinforced concrete (RC) beams.

Fiber-reinforced polymer (FRP) has been recognized as a construction material with wide applications. Apart from being manufactured in the form of bars for use as embedded reinforcements, FRP can be produced in the form of sheets externally bonded to reinforced concrete (RC) structures for the purposes of strengthening and repair [1,2]. Although some types of FRP (e.g., glass or basalt fiber-reinforced polymers) possess relatively low deformation modulus, when used as an additional externally bonded component (sheet, plate, laminate, etc.) to the RC element, the resulting composite can be effective in controlling the development of cracks [3]. The externally bonded component introduces additional failure modes to the composite structural element that should be duly considered in the design, such as brittle loss of the bond at the FRP-concrete interface [4,5,6,7,8], and rip-off of the concrete [9]. Figure 1 illustrates the different failure modes, which are characteristic of the externally bonded flexural RC members.

To enhance the crack resistance of concrete, steel fibers may be added as internally distributed reinforcement [10,11], which is referred to as steel fiber-reinforced concrete (SFRC). Recent studies [12,13,14] have proven that the addition of steel fibers could reduce the crack spacing and crack widths, and improve the serviceability performance of steel bar reinforced concrete members. Moreover, previous experimental works [15,16] have shown that the incorporation of steel fibers could improve the toughness of concrete and effectively suppress the tendency of concrete crushing, spalling, and shear failure. SFRC has been successfully utilized in infrastructure such as tunnel linings to increase the damage resistance [17,18,19], and at the same time, the amount of conventional steel bar reinforcement could be reduced [20]. With the advantages gained from using steel fibers, the structural applications of SFRC have been continuously growing. Due to aging and prolonged service, structures built with SFRC would require maintenance and rehabilitation. Therefore, the strengthening and repair of concrete structures by FRP will not be limited to conventional RC elements, but will also be inclusive of the distributed reinforcement system in SFRC elements.

Among various FRP materials, in view of the outstanding mechanical properties and consistent quality of the carbon fiber-reinforced polymer (CFRP) (as an artificial material which is manufactured under controlled processes and is not influenced by the natural inherent variability of properties), it is employed as the external sheeting material in the present research. It is envisaged by the authors that the failure mode of a composite RC beam bonded with an external CFRP sheet (or FRP sheet in general) would be heavily dependent on the bond characteristic between the fiber sheet and the concrete. In common practice, the CFRP sheets are usually bonded to the concrete surface by means of epoxy adhesives. Pilot studies have been conducted on combinations with mechanical fastening to form a hybrid connection of CFRP sheets [21]. Herein, particular attention is paid to the roles of hybrid jointing in controlling the bond failure and the ultimate failure modes of composite beams.

To obtain synergistic effects with superior performance of SFRC, carbon fiber is a potentially optimal material to be used. Hence, the behavior of composite members of SFRC bonded with external CFRP sheet warrants dedicated research. In previous experimental investigations [21,22,23], it has been revealed that the application of steel fibers altered the failure character and thus increased the load-carrying capacity of RC flexural members externally bonded with CFRP sheets. While the RC beams tended to fail by splitting of the concrete cover at the level of longitudinal reinforcement, the SFRC beams tended to fail by debonding at the interface between the concrete and the adhesive. By altering the failure character, the load-carrying capacity of SFRC beams could be increased by almost 25% (though the failure remained brittle). Besides, the CFRP sheet effectively increased the number of cracks and the energy dissipation capability of composite beam specimens before the ultimate failure [23].

Herein, a series of SFRC composite beams internally reinforced with steel bars and externally bonded with carbon fiber sheets were fabricated and tested. Two SFRC beams without external CFRP sheets were produced as reference specimens. Four beam specimens were bonded with CFRP sheets by epoxy adhesive. Another four composite beam specimens employed the hybrid jointing technique to fix the CFRP sheets, which were connected by means of adhesive in combination with additional mechanical fastenings using specially-made small-diameter steel pins. To study the crack initiation and propagation in the composite beams, the digital image correlation (DIC) method was employed to monitor the evolution of crack patterns. At this juncture, it should be noted that the research on hybrid jointing techniques would benefit the development of novel structural systems that are applicable to both new construction and structural rehabilitation, as exemplified by the hybrid bridge structures with SFRC decks bonded to FRP girders underneath [24,25,26].

## 2. Hybrid Jointing Technique

The hybrid jointing technique of connecting a FRP sheet to a concrete member was originally proposed by Arnautov et al. [21,22]. Basically, in addition to the epoxy adhesive, the FRP sheet is fixed to the concrete by additional small-diameter steel pins. To avoid stress concentrations that cause premature failure, the layout of the steel pins needs to be appropriately designed, as reflected from the preliminary modelling using a three-dimensional finite element program [3]. In this study, the steel pins were produced from grade At800 high-strength steel, which has a yield strength of no less than 800 MPa, a tensile strength of no less than 1000 MPa, and an elongation of no less than 8%.

**Figure 2:**
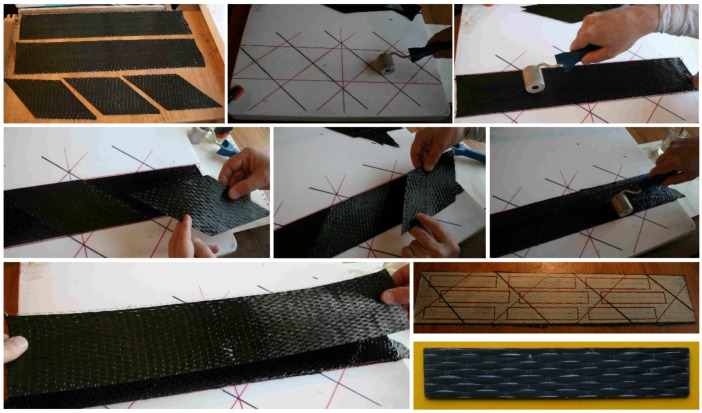
Production of the carbon fiber-reinforced polymer (CFRP) coupon specimens.

To establish the required number of steel pins for fastening the CFRP sheets while avoiding bearing failure of the CFRP and shear failure of the pins, lay-up coupon specimens were prepared and tested for the bearing response in accordance with the American Standard ASTM D5961/D5961M-01 methodology. The lay-up coupon specimen was made up of carbon fiber laminates and it had dimensions of 120 mm × 26 mm × 2 mm. The procedures of preparing the coupon specimens were as follows (Figure 2): firstly, a layer of the CFRP sheet is placed with its main direction parallel to the longitudinal direction of the coupon (0 degree), the second layer of the CFRP sheet is glued to the first layer with its main direction turned 45°, the third layer of the CFRP sheet is glued to the second layer with its main direction turned 90°, and the fourth layer of CFRP sheet is glued to the third layer in the same direction as the first layer. Finally, the carbon fiber epoxy laminates were drilled through to form holes at designated positions and fastened by steel pins. This is also referred to as a [0°/±45°/0°]_s_ lay-up coupon (Figure 2).

Without bonding to concrete as in the setting of composite beams, the coupon specimens were tested to assure both the bearing strength of the CFRP laminates and the shear strength of the steel pins. During testing, the coupon was gripped at one end and mounted to a fixture loading plate by a bolt at the other end. The assembly was loaded in tension to determine the tensile force (measured by the load cell) and the deformation (measured by the extensometer) up to failure. The tests revealed that two pins are capable of resisting the tensile stresses in the pinned joint characteristic of the debonding failure of the adhesive connection of the CFRP sheets, which were assessed in the previous study [23].

**Figure 3:**
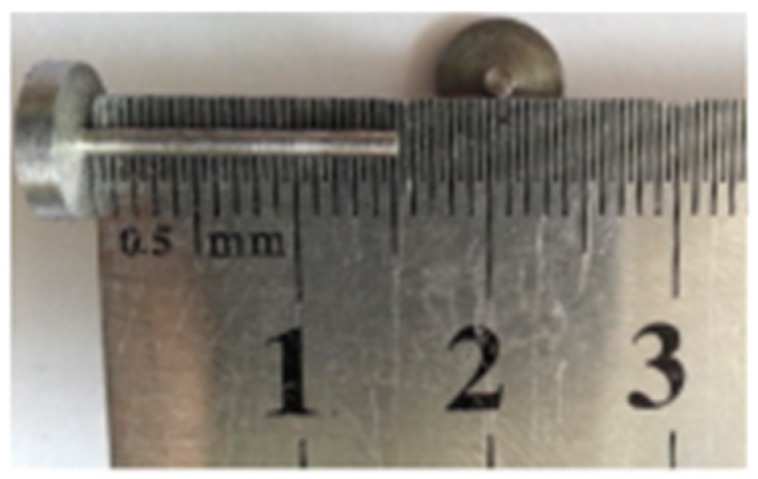
Fastening steel pins.

**Figure 4:**
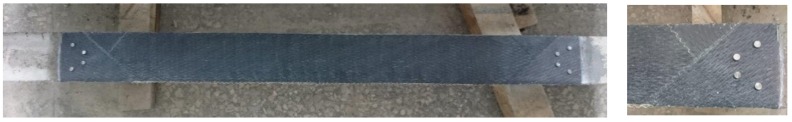
Hybrid jointing using 2 × 4 pins.

**Figure 5:**
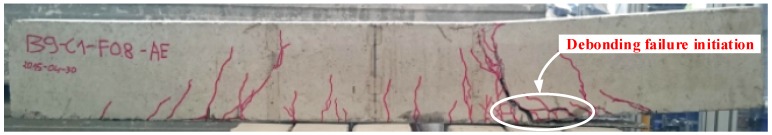
Failure of a beam with hybrid jointing using 2 × 4 pins [23].

**Figure 6:**
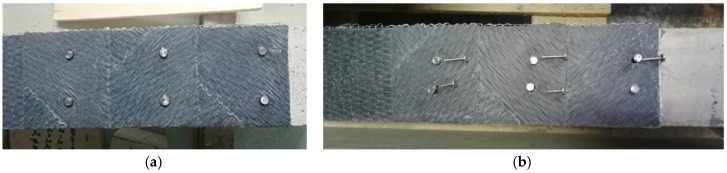
Hybrid jointing using 2 × 6 pins: (**a**) installed pins; (**b**) pins of 15 mm and 25 mm lengths.

It should, however, be noted that the actual layout of the steel pins to avoid unacceptable stress concentrations and local failure of the concrete needs to be tested with the presence of the concrete member. Besides, the bond strength between the CFRP and concrete needs to be tested with concrete members jointed with the CFRP sheets. Steel pins of 1.4 mm in diameter and 15 mm in length were fabricated, as depicted in Figure 3. Holes of 1.45 mm diameter were drilled through the carbon fiber laminates of the coupon specimens at 18 mm from the boundary edge. Designated numbers of pins from two up to six were tested. It was found that the bearing resistance of the CFRP sheets and the shear resistance of the steel pins were sufficient. In a separate study [22,23], different layouts of the pins were applied to the fastening of CFRP sheets to concrete beams. Holes of 3 mm diameter were drilled through the CFRP sheets into the concrete to enable installation of the steel pins. It was found that the use of 2 × 4 pins resulted in sudden debonding of the CFRP sheets from the beam (Figure 4), with the initiation of crack at the boundary of the pure bending zone (Figure 5). Alternatively, the use of 2 × 6 pins with the longitudinal spacing of the steel pins was taken as the experimental distance between cracks, equal to 100 mm (Figure 6), resulted in an increase of the maximum load and deformation at failure by approximately 8% and 20%, respectively. Hence, the use of a 2 × 6 array of pins was rather effective. Nevertheless, the length of the pins was found to be insufficient in suppressing the failure of the bond as the pins were forced out from the concrete. Therefore, a set of pins of 25 mm in length was fabricated for the composite beam tests.

## 3. Experimental Program

**Figure 7:**
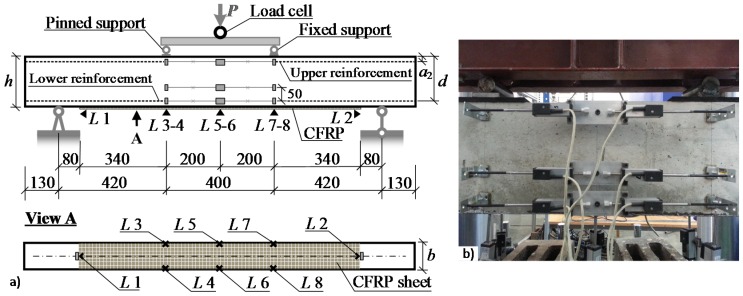
Beam tests: (**a**) geometry of the beam and monitoring scheme; (**b**) Linear variable displacement transducers (LVDT) for monitoring surface deformation.

**Figure 8:**
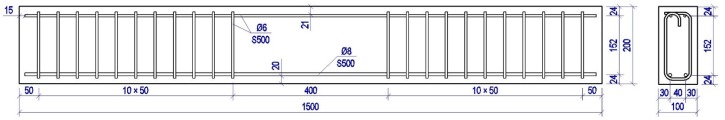
Reinforcement detailing of the beams.

**Figure 9:**
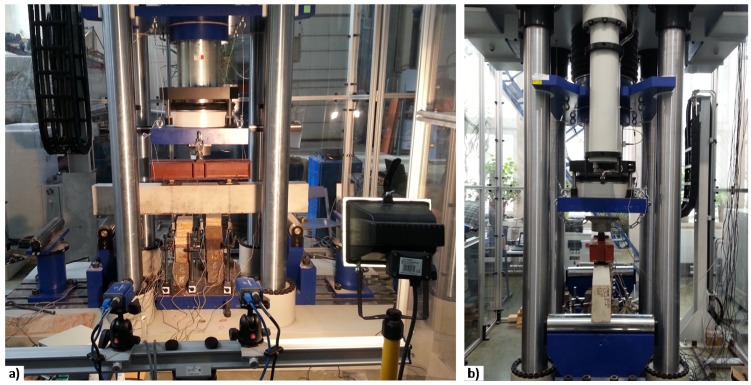
Beam tests: (**a**) test setup with high-speed cameras; (**b**) loading frame.

The experimental program encompassed 10 composite beams. All beams had a uniform cross section of 100 mm width and 200 mm depth, nominal length of 1500 mm, and span length of 1240 mm. The beams were simply supported and subjected to four-point bending with shear spans of 420 mm at both sides (Figure 7). The reinforcement detailing of the beams is illustrated in Figure 8. The tension reinforcement was 2 mm × 8 mm diameter steel bars, and the compression reinforcement was 2 mm × 6 mm diameter steel bars. To avoid shear failure, the 6 mm diameter steel stirrups were spaced at 50 mm along the shear spans. The specimens were loaded using a 5000 kN hydraulic actuator in a stiff testing frame (Figure 9). The loading rate was 0.2 mm/min.

Linear variable displacement transducers (LVDT) (*L* 3 to *L* 8 in Figure 7) were used to measure the vertical displacements in the pure bending zone. Slip of the CFRP sheets with respect to the concrete surface was measured using LVDT (*L* 1 and *L* 2 in Figure 7). To assess the average curvature in the pure bending zone, three continuous LVDT lines were located at different heights as shown in Figure 7. The upper and lower LVDT lines were placed along the top and bottom reinforcement, whereas the middle line was located 50 mm offset from the bottom one. A load cell was used to measure the applied load. All measured data from the LVDT and the load cell were collected and recorded by an Almemo 2890-9 data logger. The crack development was monitored using a high-speed camera and digital image correlation (DIC) system (Figure 9) with the aid of *DaVis 8.1.6* software by *La vision*.

All beams were made of SFRC containing 0.8% steel fibers by volume. The fibers (*Krampe Harex*) were 50 mm in length and had an aspect ratio of 50 with hooked ends. Table 1 presents the composition of the concrete, which had a design compressive strength of 50 MPa. The beams were cast in wooden formwork and were demoulded in 2 days after casting. Six ∅ 150 mm × 300 mm cylinders and seven prisms of 150 mm × 150 mm × 600 mm dimensions were produced to determine the material properties of the concrete. All specimens were stored in the laboratory at an average temperature of 21.8 °C and relative humidity (RH) of 62.5%. The mean cylinder compressive strength at 28 days of age was determined to be 58.6 MPa. The mean residual strength of SFRC was determined from the bending test of notched prisms using the RILEM (International Union of Laboratories and Experts in Construction Materials, Systems and Structures) methodology [27] and was found to be equal to 1.280 MPa and 1.342 MPa at the crack widths (taken as crack mouth opening displacement) of 0.5 mm and 3.5 mm, respectively.

For the steel reinforcement, three samples were taken from each diameter of the steel bars for undergoing the tensile test. The yield strength and elastic moduli of the 6 mm and 8 mm diameter steel bars were determined to be 565 MPa and 209 GPa, and 545 MPa and 203 GPa, respectively. Regarding the fastening steel pins, they were made of grade At800 high-strength steel, and the length and diameter were respectively 25 mm and 2.5 mm. Three samples of steel pins were tested for the ultimate tensile strength, and the average value of the test results was 1611 MPa.

**Figure 10:**
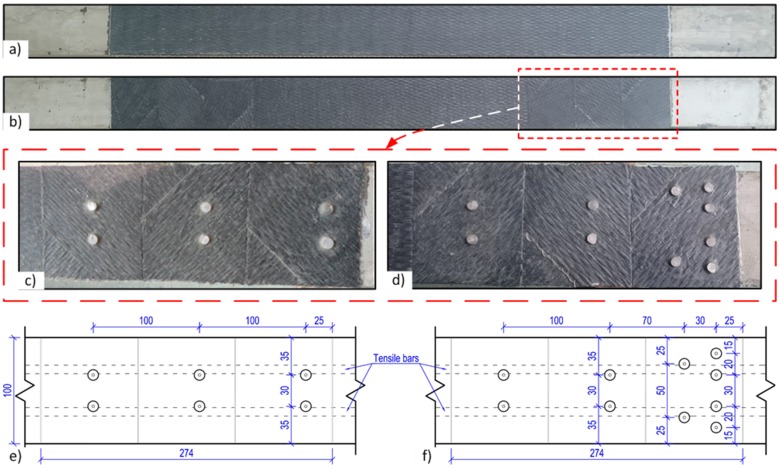
CFRP sheets: (**a**) distribution of the sheets without fastening; (**b**) with additional lay-up for hybrid jointing; (**c**) layout of the pins of beam *I(II)-B4-C-A12*; (**d**) layout of the pins of beam *I(II)-B5-C-A20*; (**e**) dimensions of 2 × 6 pins; (**f**) dimensions of 2 × 10 pins.

All beams were cast at the same time and split into two groups for bonding CFRP sheets (denoted by “*I*” and “*II*” at the beginning of the specimen numbers). Each beam in the groups was assigned a serial number (denoted by “*B1*”, “*B2*”, “*B3*”, “*B4*”, and “*B5*”). Two beams (*I-B1-Ref* and *II-B1-Ref*) without external CFRP sheets were designated as the reference specimens, whereas the remaining eight specimens were bonded with CFRP sheets at the bottom surface (denoted by “*C*” in the specimen number). The CFRP sheets were bonded to the concrete surface by epoxy adhesive (denoted by “*A0*”), or additionally fastened by steel pins in arrangements of 2 × 6 pins (denoted by “*A12*”) or 2 × 10 pins (denoted by “*A20*”) (Figure 10). The main parameters of the beams are listed in Table 2 with the notations of geometry evident from Figure 7. Other parameters presented in the table include the areas of tension reinforcement (*A_s_*_1_) and compression reinforcement (*A_s_*_2_), area of the CFRP sheet (*A_CFRP_*), the age at testing (*t*), and the number of pins in the hybrid jointing.

Uni-directional CFRP sheets *MapeWrap C UNI-AX* with the mass-per-area of 300 g/m^2^ (corresponding to 0.166 mm equivalent thickness of dry fabric) were used. The tensile strength and modulus of elasticity of the CFRP sheets were 4830 MPa and 230 GPa, respectively. These parameters were determined for the dry fabric. Since the epoxy adhesive has a relatively low modulus of elasticity [28], its contribution to the overall stiffness of the beams was neglected in further analysis. Before bonding the external CFRP sheets, the concrete surface was cleaned of any oil and dust, and levelled with the application of epoxy putty and primer. The wet lay-up system was used. The CFRP sheet was placed with its main direction along the longitudinal direction of the beam and glued to the concrete surface using two-component epoxy resin (*MapeWrap 31*). The epoxy adhesive was allowed to cure for seven days.

**Figure 11:**
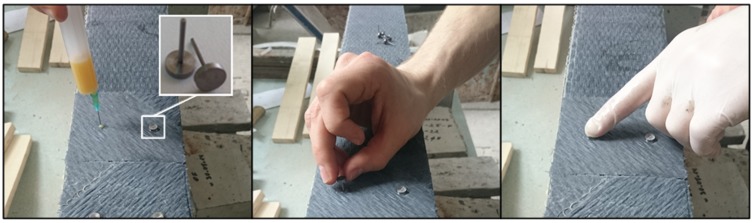
Installation of steel pins.

For the beam specimens with additional steel pin fastenings, to protect the CFRP from local failure due to stress concentration at the pins [21], the [0°/±45°]_s_ lay-up was used in the anchorage zone as shown in Figure 10. On top of the first layer of the CFRP sheet (0 degree), the second layer of CFRP sheet was glued to the first layer with its main direction turned 45°, and the third layer of CFRP sheet was glued to the second layer with its main direction turned 90°. After curing of the epoxy resin, holes of 4 mm diameter were drilled through the lay-up CFRP sheets into the concrete for installing the fastening steel pins. The steel pins were each fastened into the drilled hole using the same epoxy resin as for the sheets (Figure 11).

## 4. Test Results and Discussion

### 4.1. Flexural Stiffness

**Figure 12:**
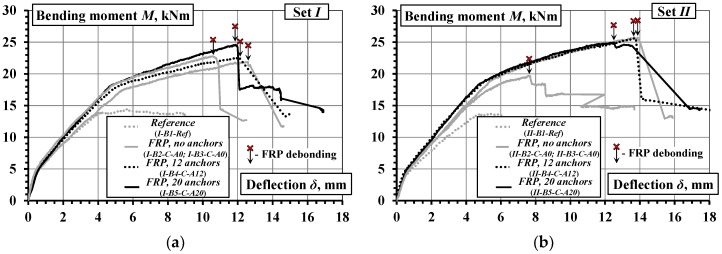
Moment-deflection diagrams of the beams: (**a**) group *I*; (**b**) group *II*.

Figure 12 shows the bending moment versus mid-span deflection diagrams of the beams. The reference beams exhibit considerably lower load-carrying capacity and smaller yielding and ultimate deflections than the remaining beams. For the specimens bonded with the CFRP sheet without additional fastenings, the deformation behavior and ultimate capacity among the beams *I-B2-C-A0* and *I-B3-C-A0* (Figure 12a), and *II-B2-C-A0* and *II-B3-C-A0* (Figure 12b) established considerable variations. For the specimens with the adoption of hybrid jointing, i.e., beams *I-B4-C-A12* and *I-B5-C-A20* (Figure 12a) and *II-B4-C-A12* and *II-B5-C-A20* (Figure 12b), the load-carrying capacity was marginally higher and the ultimate deflection was substantially larger.

Local effects in deflection measurements might have a significant influence on the assessment of flexural stiffness of composite elements [29]. At the elastic stage, the error is almost linear (i.e., the error increases approximately proportionally with the deflection magnitude) up to the first crack. It can be eliminated by accounting for the elastic deformations at the supports [27]. In the post-cracking regime, the deflection measurement errors are related to the distribution (and number) of the flexural cracks. The error is influenced by the size effect of the specimen. It would generally decrease by increasing the beam dimensions and the load-carrying capacity of the specimen.

**Figure 13:**
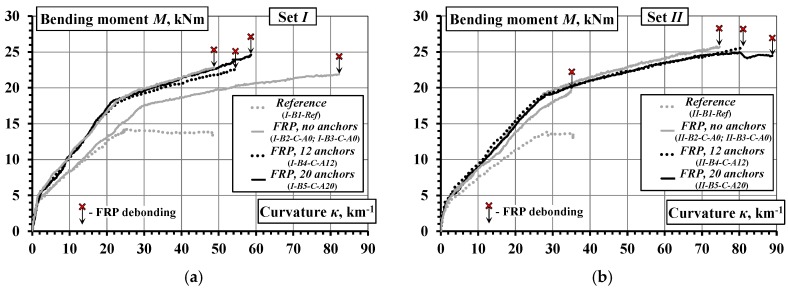
Moment-curvature diagrams of the beams: (**a**) group *I*; (**b**) group *II*.

To reduce the scattering of the results, the flexural stiffness is assessed with the help of the moment-curvature diagrams determined using the surface strains averaged along each of the LVDT lines. A recent study [30] has revealed that such a technique is efficient for the comparative analysis of beams with composite reinforcement and different flexural rigidity (as characteristic for this experimental program). Following the methodology detailed in reference [31], the curvature averaged over the pure bending zone is calculated as:

(1) $$\kappa =\frac{1}{3}\sum_{l=2}^{3}\sum_{k=1}^{l-1}\frac{D_{k}-D_{l}}{h_{kl}},$$

where *D_k_* and *D_l_* are the averaged strains along the *k* and *l* gauge lines (Figure 7), and *h_kl_* is the distance between the lines (*k*, *l* = 1 … 3, *k* ≠ *l*). Figure 13 shows the moment-curvature diagrams obtained.

Unlike the mid-span deflection diagrams (Figure 12), the moment-curvature graphs demonstrate reduced scattering, as shown in Figure 13. The gradient of the moment-curvature curves represents the flexural stiffness of the composite beams. The divergence between the moment-curvature curves could be attributed to the different geometry: the effective depth *d* and the height *h*, which affects the lever arm of the reinforcement in resisting flexure. An important property of the moment-curvature relationships is that these averaged diagrams can be used for inverse analysis, i.e., derivation of the tension-stiffening model based on the smeared cracking approach [32]. Application of the inverse analysis is presented in Section 4.3.

### 4.2. Cracking Behaviour

**Figure 14:**
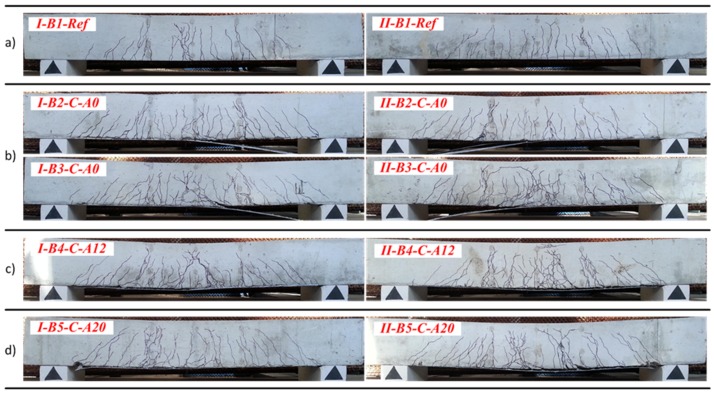
Crack pattern of the beams: (**a**) reference; (**b**) with pure adhesive bond; (**c**) with 2 × 6 pins; (**d**) with 2 × 10 pins.

**Figure 15:**
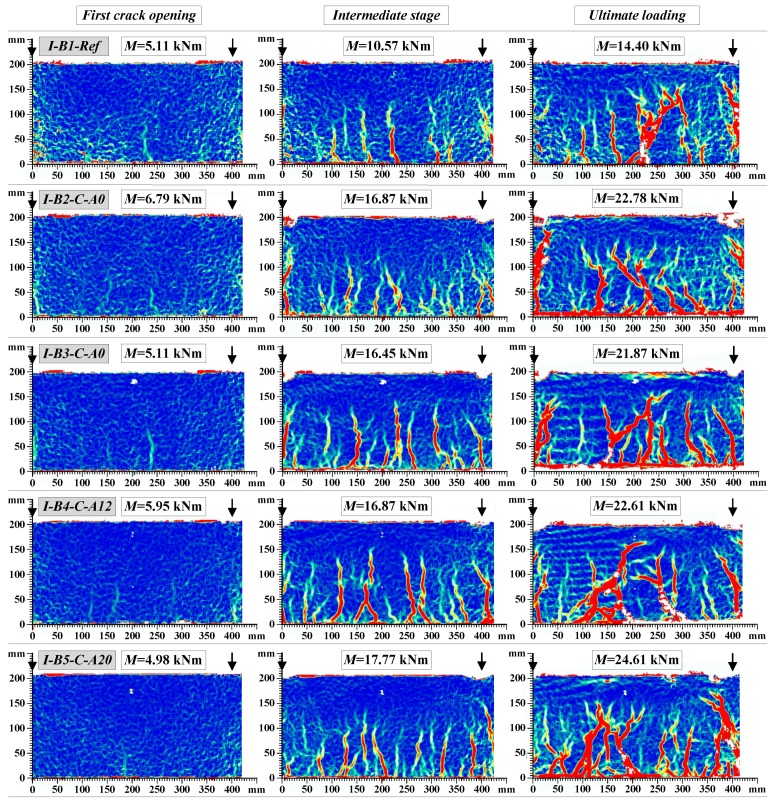
Development of cracks in the group *I* beams.

**Figure 16:**
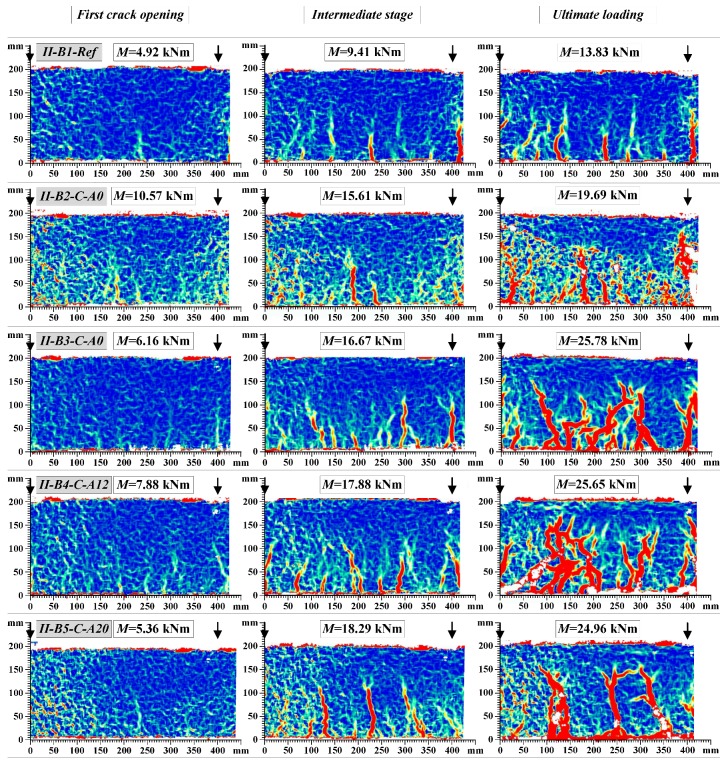
Development of cracks in the group *II* beams.

Figure 14 shows the final crack pattern of the beams. The multiple cracks smeared-out in the tensile zone indicated the efficiency of the composite members: the external sheet transferred the tensile stresses through the crack planes and suppressed the localization of the discrete cracks. In comparison with the unbonded counterparts, the externally bonded beams were characterized by the smeared crack pattern through the bonded zone; and the major cracks were formed only at relatively high levels of load approaching the yielding of the steel reinforcement. As can be observed in Figure 14b, the peeling-off failure is typical for the externally bonded beams with pure adhesive bonding of CFRP sheets, while the hybrid jointing assured connection of the external sheet at the ultimate loading stages (Figure 14c,d). Further elaboration of the load resisting mechanisms will be presented in Section 4.4. Figure 15 and Figure 16 show the development of cracks in the pure bending zone monitored with the DIC system for group *I* and group *II* composite beams, respectively. The images were mirrored backside views of the crack patterns shown in Figure 14. The development of cracks at the first crack opening, intermediate stage, and ultimate loading are depicted.

It is evident from the crack patterns that the externally bonded CFRP sheet, whether fixed by the hybrid jointing technique or not, was effective in distributing a few numbers of large cracks in the reference beams to large numbers of finer cracks with reduced crack spacing. Therefore, at the same level of flexural deflection and curvature, the crack widths of beams bonded with CFRP sheets would be less than that of the reference beams. Similar results were obtained for the beams externally bonded with basalt fiber sheets [33]. That indicates that the efficiency of external reinforcement sheets in spreading the crack widths significantly improved the serviceability performance of the composite beams.

### 4.3. Residual Strength of the Cracked Concrete

**Figure 17:**
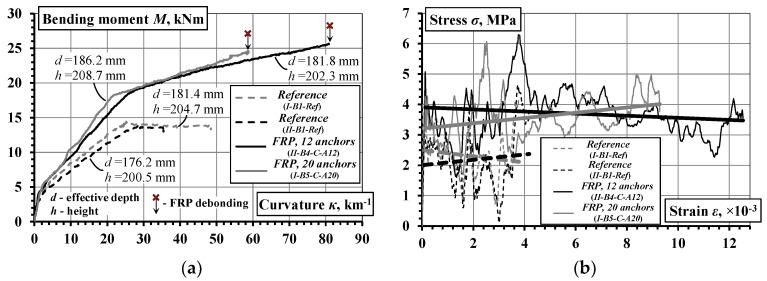
Comparative analysis of the flexural stiffness of the selected beams: (**a**) moment-curvature diagrams; (**b**) residual stresses of tensile concrete.

In order to reduce the effect of geometry on the flexural stiffness, the average stress–average strain diagrams of the tensile concrete are analyzed using the inverse technique [32]. The inverse analysis is based on the following approaches and assumptions: (1) the smeared crack approach (i.e., the cracking of concrete is accounted for through the constitutive law of concrete); (2) a linear strain distribution within the section depth; (3) all concrete layers in the tension zone follow a uniform stress-strain law. Using the moment-curvature diagrams presented in Figure 17a, the stress-strain relationships are derived as depicted in Figure 17b. The stress-strain diagrams consist of two parts: the ascending (very steep and thus barely visible) and the descending branches. As the post-cracking behavior is of particular interest, the trend-lines for the descending branches (Figure 17b) were determined. Three key phenomena could be inferred from the stress-strain relationships:

- Although the source curves were different, the resultant stress-strain diagrams of the composite beams are practically equivalent: the trend-lines practically coincide. The oscillations in the stress-strain diagrams could be attributed to the sensitivity of the inverse procedure to the cracking process in the beams with relatively small size (refer to [32] for a more detailed description of this issue).
- The inversely determined diagrams consist of the genuine stresses corresponding to the tension-stiffening effect and the residual stresses due to fiber interaction with the concrete. Earlier investigations of RC bending members [34,35] have shown that the tension-stiffening effect practically disappears at the tensile deformation of 2000–2500 micro-strains, which was suggested by Gribniak et al. [34] as the limit for quantifying the residual stresses of SFRC. The latter parameters were experimentally determined using the RILEM methodology [27] (details have been reported in Section 3) as corresponding to 31.1% and 32.7% of the theoretical tensile strength of concrete (assessed per Eurocode 2, based on *f_ctm_* = 4.11 MPa) at the crack mouth opening displacement of 0.5 mm and 3.5 mm, respectively. However, the trend-lines indicate an almost *constant* portion of tensile stresses associated with the cracked concrete. In relative terms, the respective average stresses of the reference and the externally bonded beams represent 54.7% and 88.9% of the strength *f_ctm_*. In fact, these values reflect the overall tension-stiffening effects consisting of the components of internal and external reinforcement. Thus, taking into account the effect of tension-stiffening, the residual strength is not only a material property of SFRC, it is associated with the characteristics and amount of the structural reinforcement.
- The ultimate strains of the stress-strain diagrams are quite different. Accounting for the theoretical cracking strain of the concrete (assessed per Eurocode 2, *ε_cr_* = *f_ctm_*/*E_cm_* = 0.109 mm/m), the ultimate normalized strain in the reference beams, being dependent on the yielding strain of the steel reinforcement, did not exceed 40*ε_cr_*. Specimens with external CFRP sheets possessed the tensile strain over 70*ε_cr_* (not shown in Figure 17b), whereas additional mechanical fastenings increased the ultimate deformations up to 125*ε_cr_*. The observed reduction of the ultimate deformation (90*ε_cr_*) in the beam with 2 × 10 pins (Figure 17b) could be related to the localized failure in the anchorage zone. This effect is discussed in the next section.

### 4.4. Structural Integrity

**Figure 18:**
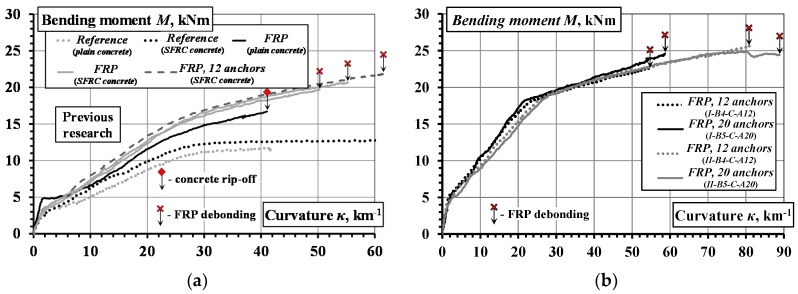
Moment-curvature diagrams: (**a**) previous test results [23]; (**b**) beams with hybrid jointing of CFRP sheets.

The structural integrity is understood as a property to resist the applied loading without failure or loss of the connection between the composite components. This is a general issue in the design of composite structural elements [36]. The present study is focused on the composite action of the external CFRP sheets and concrete in the tension zone of the beams. The current results are to be considered in the context of the previous findings [21,22,23]. The structural integrity could be related to the ability of steel fibers to alter the failure character, resulting in an increase of the load-carrying capacity of the beams with external CFRP sheets. Figure 18a presents the moment-curvature diagrams of the beams from previous research [23]. The following three important observations could be made:

- The fibers had a limited effect on the flexural stiffness, nevertheless they are very effective in preventing brittle failure of the concrete.
- Debonding of the CFRP sheets caused brittle failure of the composite beams made of SFRC.
- Mechanical fastenings increased the ultimate deformations (ductility) of the composite beams.

Regarding the role of steel fibers, the effects of the addition of fibers with different geometries and aspect ratios on the mechanical properties of SFRC might vary significantly [37]. Furthermore, the orientation of the fiber significantly affects the isotropy, homogeneity, and hence the mechanical properties of SFRC. The relationship between construction methods and fiber orientation, as well as accurate and reliable test methods of fiber orientation are yet to be determined, despite years of research [38,39,40,41,42]. Therefore, further research aimed at the selection of the proper type and proportion of steel fibers and allowing for the effects of construction methods on fiber orientation is recommended.

The hybrid jointing technique results in controlled load-carrying capacity and enhanced deformation capacity. Figure 18b presents moment-curvature diagrams of four beams with the external CFRP sheets fixed using additional fastening steel pins. It is evident that the flexural stiffness amongst these elements is similar though their ultimate deformations are quite different.

**Figure 19:**
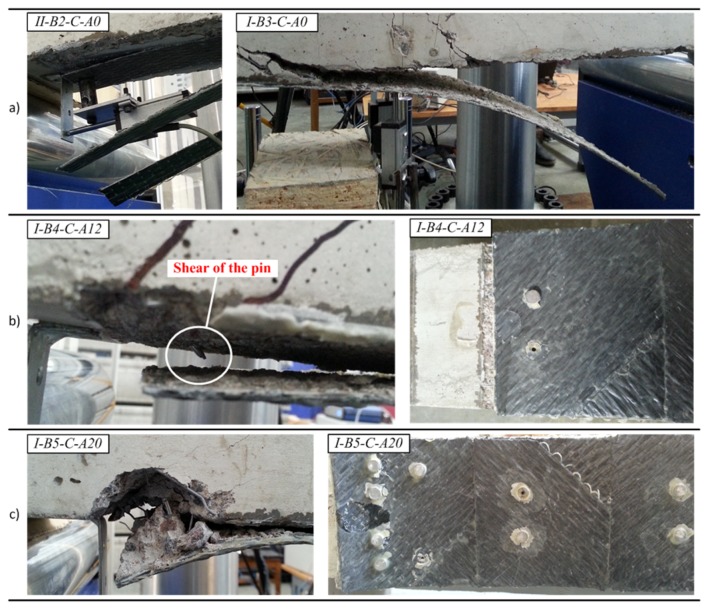
Failure character of the beams: (**a**) sudden peeling-off of CFRP sheets; (**b**) plastic delamination of the CFRP sheet fastened with 2 × 6 pins; (**c**) localized failure of the beam using 2 × 10 pins.

The failure character of the beams with external CFRP sheets is illustrated in Figure 19. In coherence with the previous results [23], the failure of the beams with pure adhesive bonding of carbon fiber sheets was brittle as a consequence of sudden debonding of the CFRP sheets (Figure 19a). Furthermore, a local split of the sheet was observed in the beam *II-B2-C-A0* which was the consequence of different strength of the adhesive observed after the test within the damaged bond area. The layouts of pins tested provided two distinct outcomes. As shown in Figure 19b, the arrangement of 2 × 6 pins led to shear failure of the steel pins; whereas by increasing the number and concentration of pins in the boundary zone (Figure 10), the arrangement of 2 × 10 pins caused local failure of the concrete (Figure 19c), though the failure remained ductile as the remaining pins distributed outside the localized failure zone were still capable of resisting slip of the CFRP sheet.

**Figure 20:**
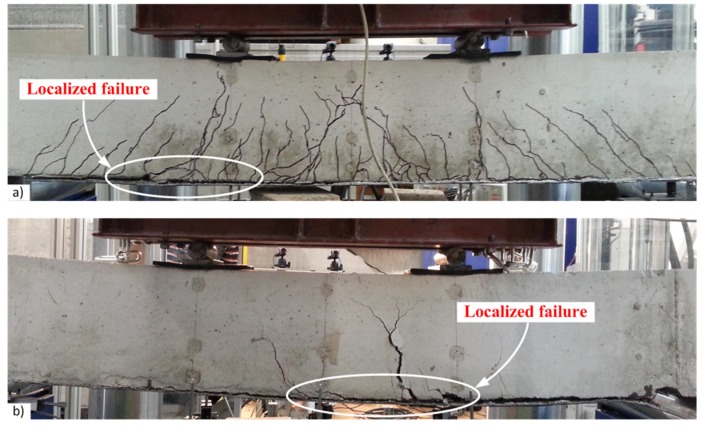
Bond failure localized near flexural cracks: (**a**) beam *I-B4-C-A12*; (**b**) beam *II-B5-C-A20*.

Monitoring results of the slip of the CFRP sheets by way of LVDT *L* 1 and *L* 2 are summarized in Table 3. As can be observed, the beams without hybrid jointing (*I-B2-C-A0*, *I-B3-C-A0*, and *II-B2-C-A0*) exhibited lower values of bending moment at the initiation of slip (the test recording for the beam *II-B3-C-A0* was lost due to failure of the LVDT connections). The beams with hybrid jointing (*I-B5-C-A20*, *II-B4-C-A12*, and *II-B5-C-A20*) exhibited the highest values of maximum slip, though contrasting results were observed for the beams *II-B2-C-A0* and *I-B4-C-A12*. Splitting of the CFRP sheet was observed in the beam *II-B2-C-A0*, and was the consequence of the high maximum slip obtained. The observed relatively small value of the maximum slip of the beam *I-B4-C-A12* can be related to the localized debonding near the flexural cracks (Figure 20a), while the pins assured the mechanical contact in the anchorage zone. For all the CFRP bonded beams in group *I* and group *II*, there was an increase in the load-carrying capacity ranging from 51.9% to 70.8% and from 42.4% to 86.0% compared to the reference beams *I-B1-Ref* and *II-B1-Ref*, respectively.

The hybrid jointing of the CFRP sheets is characterized by the improved ductility: the ultimate deformations were increased by 20% on average. The failure process became gradual: the debonding was localized near a critical crack within the pure bending zone (Figure 20) while the pins were resisting the shear stresses. The collapse of the external bond was preceded by an evident deformation of the pins. Nevertheless, the layout of the pins has to be designed properly as a concentration of the pins may localize the failure of concrete.

## 5. Summary and Conclusions

The present experimental study has investigated the structural integrity of SFRC composite beams reinforced with internal steel bars and external CFRP sheets. The flexural stiffness, cracking, and failure mechanisms of 10 beam specimens have been evaluated. In view of the strong dependence of the failure behavior of SFRC beams on the bond properties of the CFRP sheets, hybrid jointing has been utilized to control the debonding. The additional mechanical fastening involved the use of 2.5 mm diameter steel pins in arrangements of 2 × 6 pins and 2 × 10 pins. The study has revealed that:

1. The external sheets, by transferring stresses through the crack planes, were effective in suppressing the localization of the discrete cracks. The final crack pattern manifested multiple cracks smeared in the tensile zone.
2. The inverse analysis of the moment-curvature response indicated a constant level of tensile stress associated with the cracked concrete. In relative terms, the respective average stresses of the reference and the externally bonded beams represent 54.7% and 88.9% of the theoretical tensile strength of the concrete *f_ctm_* assessed per Eurocode 2, and these values reflect the tension-stiffening effects which consist of the components of internal and external reinforcement. In other words, the simplified tension-stiffening model (with a constant residual strength of the cracked concrete ≈ 0.9*f_ctm_*) is adequate for representing the deformation behavior of the beams with the external CFRP sheets.
3. Accounting for the theoretical cracking strain of the concrete assessed per Eurocode 2, the ultimate normalized strain in the reference beams, being dependent on the yielding strain of the reinforcing steel, did not exceed 40*ε_cr_*, whereas for the specimen with external CFRP sheets, the tensile strain was over 70*ε_cr_*. The additional mechanical fastening increased the ultimate deformations up to 125*ε_cr_*.
4. The hybrid jointing increased the ultimate deformations (ductility) of the composite beams by 20% on average. Furthermore, the failure process became gradual and visually apparent.

From the research findings, the use of SFRC in conjunction with external CFRP sheets with hybrid jointing to form a new composite beam system is structurally efficient and robust. In general applications of the hybrid jointing technique, the layout of the steel pins has to be designed properly because a concentration of the pins may localize the failure of concrete. Further research on the optimum layout of the steel pins is needed. Besides, as the properties of SFRC are dependent on the fiber type, content, and orientation, further studies aimed at the selection of the optimum type and proportion of steel fibers and allowing for the effects of construction methods on fiber orientation is necessary.

## Figures and Tables

**Table 1:** Mix proportions of steel fiber-reinforced concrete (SFRC).

Material	Amount (kg/m^3^)
Sand 0/4 mm	865 ± 1%
Crushed aggregate 4/8 mm	404 ± 2%
Crushed aggregate 8/11 mm	606 ± 2%
Cement CEM IIA-LL 52.5 R	300 ± 0.5%
Water	150 ± 3%
Superplasticizer *MC-PowerFlow 3100*	4.0 ± 1%
Steel fibers *Krampe Harex*	65

**Table 2:** Main characteristics of the composite beams.

Group	Beam	*h* mm	*d* mm	*a*_2_ mm	*b* mm	*A_s_*_1_ mm^2^	*A_s_*_2_ mm^2^	*A_CFRP_* mm^2^	*t* Days	Layout of Pins
*I*	*I-B1-Ref*	204.7	181.4	23	103.4	100.5	56.5	–	68	–
	*I-B2-C-A0*	200.4	181.1	24	100.8	100.5	56.5	16.6	69	–
	*I-B3-C-A0*	196.0	172.5	23	101.7	100.5	56.5	16.6	70	–
	*I-B4-C-A12*	200.8	179.3	23	101.8	100.5	56.5	16.6	69	2 × 6
	*I-B5-C-A20*	208.7	186.2	24	99.8	100.5	56.5	16.6	73	2 × 10
*II*	*II-B1-Ref*	200.5	176.2	23	100.2	100.5	56.5	–	83	–
	*II-B2-C-A0*	197.0	171.6	24	101.6	100.5	56.5	16.6	87	–
	*II-B3-C-A0*	199.6	175.6	23	102.2	100.5	56.5	16.6	88	–
	*II-B4-C-A12*	202.3	181.8	24	97.5	100.5	56.5	16.6	89	2 × 6
	*II-B5-C-A20*	201.1	177.4	23	100.8	100.5	56.5	16.6	89	2 × 10

**Table 3:** Slip of the external CFRP sheets.

Beam	Number of Pins	Slip Initiation Moment (kNm)	Failure Moment (kNm)	Maximum Slip (mm)
*I-B1-Ref*	–	–	14.40	–
*I-B2-C-A0*	–	8.79	22.71	0.43
*I-B3-C-A0*	–	8.01	21.87	0.23
*I-B4-C-A12*	2 × 6	11.44	22.46	0.36
*I-B5-C-A20*	2 × 10	16.06	24.60	1.50
*II-B1-Ref*	–	–	13.83	–
*II-B2-C-A0*	–	3.62	19.69	1.00
*II-B3-C-A0*	–	–	25.72	–
*II-B4-C-A12*	2 × 6	13.09	25.61	2.32
*II-B5-C-A20*	2 × 10	10.70	24.46	1.03
